# Suicide among adults aged 30–49: A psychological autopsy study in Hong Kong

**DOI:** 10.1186/1471-2458-8-147

**Published:** 2008-05-01

**Authors:** Paul WC Wong, Wincy SC Chan, Eric YH Chen, Sandra SM Chan, YW Law, Paul SF Yip

**Affiliations:** 1 Hong Kong Jockey Club Centre for Suicide Research and Prevention, University of Hong Kong, Pokfulam, Hong Kong SAR, P.R. China; 2 Centre for the Advancement of University Teaching, University of Hong Kong, Pokfulam, Hong Kong SAR, P.R. China; 3 Department of Psychiatry, University of Hong Kong, Pokfulam, Hong Kong SAR, P.R. China; 4 Department of Psychiatry, The Chinese University of Hong Kong, Shatin, Hong Kong SAR, P.R. China; 5 Department of Social Work and Social Administration, University of Hong Kong, Pokfulam, Hong Kong SAR, P.R. China

## Abstract

**Background:**

A surge in suicide rates in middle age people in Hong Kong and many Asian countries was recently observed. However, there is a paucity of suicide research on this subgroup of people in Asia.

**Methods:**

The next-of-kin of 85 suicide cases and 85 community subjects aged 30–49 years were interviewed by a psychological autopsy approach. Information was triangulated by interview notes, coroner's court files, and police investigation reports.

**Results:**

A multiple logistic regression analysis identified the following risk factors for suicide among the middle age people in Hong Kong: the presence of at least one psychiatric disorder (OR = 37.5, 95% CI 11.5–121.9, p < 0.001), indebtedness (OR = 9.4, 95% CI 2.2–40.8, p < 0.01), unemployment (OR = 4.8, 95% CI 1.3–17.5, p < 0.05), never married (OR = 4.2, 95% CI 1.1–16.3, p < 0.05), and lived alone (OR = 3.9, 95% CI 1.2–13.4, p < 0.05).

**Conclusion:**

The data show that socio-economical factors had a strong impact on suicide in the target group. Further research is needed to explore any positive qualities that protect the middle-aged from suicide. The prevention of suicide in the middle-aged requires multiple strategies.

## Background

There are growing concerns about the recent increasing rates of middle-aged suicide in Asian countries [1,2]. The trend has also been clearly evident in Hong Kong since the sovereignty handover in 1997. In the last decade, Hong Kong was affected by a number of unanticipated socio-cultural and economical crises. Alongside the region-wide financial turmoil in late 1997, the internet dot-com bubble in late 2000, and the SARS epidemic outbreak in early 2003, an upward trend of a number of ill mental health indicators was evident by an increased demand of psychiatric and hotline services [3]. The emergence of these crises was also paralleled by a surge in suicide rates in all ages in Hong Kong. Hong Kong's suicide rate increased from 12.1 per 100,000 in 1997 to 18.6 per 100,000 in 2003 [2]. Of these increased number of suicide cases a marked increase in fatal suicidal incidents was observed in the 30-to-49-year-olds. The number of fatal suicide cases in this subgroup increased from 294 (10.4 per 100,000) in 1996 to 484 (18.9 per 100,000) in 2003 – an increase of 65% [2].

Middle-aged suicide was recognized as a major public health problem in Hong Kong [2]. However, previous studies on the local suicide problem have focused mainly on the youth and elderly subgroups [4-7]. Relatively little is known about the factors leading to suicide in the middle-aged. From Western evidence we know that correlates of suicidal behaviour vary across the life span [8]. It is therefore necessary to understand the risk factors for suicide in the middle-aged in order to inform the design of suicide prevention strategies for this subgroup of the population. In this study, we used a psychological autopsy approach, matching suicide cases with community live subjects to investigate the extent to which psychiatric, psychological, socio-economical, and life adversities might have an effect on the decision of taking one's life for the middle-aged group.

## Methods

### Study population

Subjects of this study included those who aged between 30 and 49 years extracted from the sample of a recent research study on suicide in Hong Kong [9]. Subjects in this study comprised 56.6% of the sample in the original study. Similar to most case-control psychological autopsy studies, we selected a control group of age and gender matched live community sample and interviewed their next-of-kin [10-13]. Details of the research methodology and measurements of the study were reported elsewhere [9]. This study was approved by the Institutional Review Board of the University of Hong Kong/Hospital Authority Hong Kong West Cluster (HKU/HA HKW IRB), and the Ethics Committee of the Department of Health, Hong Kong SAR.

### Procedures

#### Suicide cases

**Figure 1:**
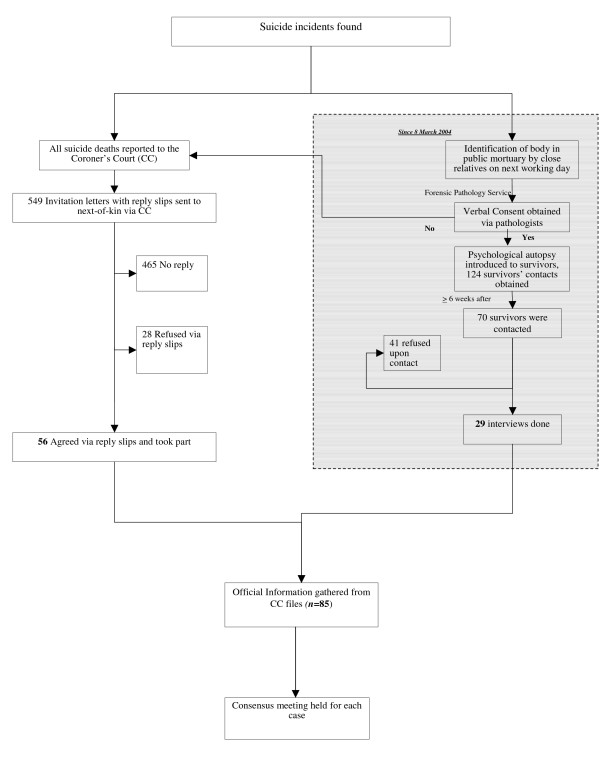
Deceased case recruitment procedures through the Coroner's Court and the referrals from forensic pathologists at public mortuaries (*n *= 85).

Figure 1 shows the procedures taken to identify suicide subjects. We identified potential subjects in the study via the Forensic Pathology Service and the Coroner's Court. A total number of 85 cases were examined; 29 suicide cases were recruited from the Forensic Pathology Service, and 56 cases were recruited through the Coroner's Court. The successful rate via the Forensic Pathology Service thus was 41.4% (29/70) and via the Coroner's Court was 10.2% (56/549). The most common reasons for not participating in the interview included the reluctance to talk about the death and lack of time.

#### Control cases

Information on a one-to-one matched community sample of controls was collected by interviewing the next-of-kin. The sample pool for the control group consisted of four hundred and forty individuals from another randomly selected sample of the general population [14]. Eighty-five control subjects, matched for age and gender with that of the suicide cases were selected for the study. The next-of-kin of the control subjects were interviewed to give proxy information on the same set of questions (excluding the circumstances of death and bereavement sections) used on the next-of-kin of the suicide cases.

#### Instruments

We classified the examined factors into four broad domains: socio-demographics characteristics, psychiatric, family history, and psychological variables. Most of the measurements were comparable with psychological autopsy studies conducted by Conwell and colleagues [15] in Rochester, the United States, by Hawton and colleagues [16] in Oxford, the United Kingdom, and Phillips and colleagues [17] in Beijing, China. All adapted measurements were back-translated and tested in a pilot study for their readability. Six next-of-kin of suicide cases participated in the pilot study. All measurements were administrated in Cantonese.

Socio-demographics characteristics investigated included marital status, educational status, ethnicity, living arrangement, employment status, and income. Psychiatric factors investigated including presence of psychiatric disorders at the time of death, history of psychiatric disorders, previous psychiatric treatment, previous suicide attempts, presence of chronic illnesses and physical disability using the SCID-I-DSM-IV-TR (Structured clinical interview for Diagnostic and Statistical Manual of Mental Disorders – Fourth Edition – Text Revision – Axis-I disorders, American Psychiatric Association, 2000). The SCID-I-DSM-TR is the standard measurement of psychiatric disorders in psychological autopsy study [18]. The Impulsivity Rating Scale [19], and Social Problem-Solving Inventory [20] were used to assess psychological conditions. A culturally specific scale on healthy living styles was developed to measure five aspects of healthy living: healthy diet, physical activities, emotional expressions, interests and habits, and continual learning. The five items of the healthy living scale exhibited satisfactory internal consistency (Cronbach alpha = 0.62). Social factors including social isolation and culturally specific adverse life events variables including relationship, family, work, physical health, and legal issues were investigated.

#### Interviews and coroner's court files

The interviews of the suicide cases were carried out at an average of 7.3 (*SD *= 4.0) months after the death incident. After obtaining information on the decedents, the informants' responses to bereavement were also collected and reported elsewhere [21]. The majority of the informants were the spouses (n = 28, 32.9%) and siblings (n = 29, 34.2%) of the deceased, 9 (10.6%) were children, 9 (10.6%) were parents, and 10 (11.8%) were others including friends, romantic partners, relatives, and co-workers. The interviews lasted from 1.5 hours to 6 hours (*M *= 3.4 hours). Should grieving informants be uniquely anguished or angry, an emergency protocol was prepared for the safety of the informants or researchers. An emergency light switch at the interview room was installed in order to provide help and support for interviewers during emergency situation. Should an informant report distress during and/or after the interview, brief counselling would be provided by the researchers (PWCW and SSMC). When necessary, distressed informants would be referred for psychological and psychiatric support. Nine informants required brief counselling and referral information. The interviews of the control cases lasted from 1 hours to 1.5 hours. The majority of the informants were spouses (n = 48, 56.5%), children (n = 15, 17.6%), siblings (n = 14, 16.5%), and 8 (9.4%) were others. No subjects from the control group required any counselling services from the researchers.

We also reviewed Coroner's files to supplement and validate the information provided by the informants. The files contained demographic and background information; circumstances of the death; life situations; autopsy and toxicology reports; witness statements; police investigation records; medical and psychiatric reports; suicide notes; and insurance policies (if any).

### Data analysis

SPSS-PC software version 12.0 was used for statistical analyses (SPSS Inc, Chicago, IL, USA). Before investigating the individual factors of suicide, the age and gender distributions and the means of suicide between the 85 suicide cases and all suicides aged 30–49 in 2003 (*N *= 484) were compared in order to assess the representativeness of the sample. The demographic characteristics of the suicide samples and controls were also compared. Binary logistic regression analyses were then employed to obtain the unadjusted odds ratios and their 95% confidence intervals for each independent variable. Significant variables (with *p *< 0.001) were then entered into a multivariate logistic regression model to investigate the more robust risk factors of suicide. A forward and backward stepwise elimination method was used in order to identify a stable model. The problem of multi-collinearity of the final model was also tested but was found to be insignificant: the minimum tolerance statistic for the independent variables in the final model was 0.85 and variance inflation factors (VIF) was small, with a maximum of 1.18, while a tolerance value below 0.10 and a VIF above 10 denote high collinearity among variables [22].

## Results

### Demographics

The 85 subjects were representative of the overall middle-age suicide (*N *= 484) in terms of gender, age, and method of suicide. There was no significant difference in the age distribution between the sample and all suicides aged 30–49 in 2003, either in males (*M *= 38.68; *SD *= 5.39; *p *= 0.06) and in females (*M *= 40.88; *SD *= 5.94; *p *= 0.27). The male to female ratio in the sample was 1.66 whereas that in all suicides aged 30–49 in 2003 was 2.04. Comparing the most common principle methods used in the deceased group and in all suicides aged 30–49, no significant difference was found in those died by jumping (*p *= 0.95), charcoal burning (*p *= 0.42), and hanging (*p *= 0.51). However, the proportion of females who committed suicide by charcoal burning in the suicide subjects was lower than the suicide cases in 2003.

The three most common methods of suicide used by the subjects were charcoal burning in 40 (47.1%) cases, jumping from height in 33 (38.8%) cases, and hanging in 10 (11.8%) cases. The majority (n = 69, 81.2%) of the subjects died at home, six (7.1%) in a public area, four (4.7%) at a relative or friend's residence, three (3.5%) at workplace, and three (3.5%) at other locations. Thirteen (15.3%) of the subjects died by suicide on a special day, i.e. anniversaries, birthdays, or festivals. According to the informants, 13 (15.2%) of the victims completed the death within 24 hours of their initial suicidal thoughts, 12 (14.2%) from one to seven days, eight (9.4%) within a week and a month, seven (8.2%) from one to two months, and 22 (25.9%) thought about committing suicide two months or more before the actual act. The informants of 23 (27.1%) cases were not able to provide this information. Thirty-four (40.0%) out of 85 cases left at least one suicide note in the form of message, letter, or diary, and 39 (45.9%) of them either implicitly or explicitly expressed their suicidal plan.

### A binary logistic regression

Tables 1 and 2 show that the suicide group and control group differed significantly on a wide range of socio-demographic, and psychological and psychiatric factors, respectively. The risk of suicide increased substantially as certain risk factors became more pronounced, namely being never married; living alone; unemployed; low income; presence of unmanageable debt; high level of expressed emotions at home; presence of psychiatric diagnosis; and impulsive (all *p *< 0.001). In contrast, social support including a wider social network and more accessible support within the network, and those who acquired social problem solving skills and led a healthy lifestyle were at less risk of dying by suicide (*p *< 0.001). Psychiatric illnesses were more frequent among the suicides than in the controls. Mood disorders were the most prevalent, presented one in two of the suicides (50.6%) and one in ten of the control subjects. Psychotic disorders were the second most prevalent psychiatric disorders among the suicide subjects (21.2%), followed by pathological gambling (14.1%), substance use (7.1%), adjustment disorder (8.2%), and anxiety disorders (4.7%). In the control group, only 11 controls had a diagnosed psychiatric disorder (12.9%) and 10 of them had mood disorders. Comorbidities of psychiatric disorders were found in 26 (30.6%) suicides but in one (1.2%) control subject only. It is important to note that previous attempt was found in 43 (50.6%) suicide victims but none of the control subjects.

**Table 1:** Comparison of sociodemographic characteristics of suicides and controls and unadjusted parameter estimates^a^

	Cases (N = 85) n(%)	Control (N = 85) n(%)	Odds Ratio (95% CI)^b^
**Socio-demographics Conditions**			
Marital Status ^1^			
Currently married	50(58.82)	67(78.82)	1
Separated	9(10.59)	8(9.41)	1.51 (0.54–1.51)
Never married	26(30.59)	10(11.76)	3.48 (1.54–3.48)**
Educational Level ^2^			
Above Form 3	36(42.35)	51(60.00)	1
Form 3 or below	49(57.65)	34(40.00)	2.04 (1.11–2.04)
Living Arrangement ^3^			
Lived with someone	67(78.82)	61(71.76)	1
Living alone	18(21.17)	24(28.23)	3.46 (1.83–3.46)**
Employment Status ^4^			
Full-time employed	33(38.82)	65(76.47)	1
Unemployed or underemployed	45(52.94)	13(15.29)	6.82 (3.23–6.82)**
Economically inactive	6(7.06)	3(3.53)	3.94 (0.93–3.94)
Monthly Income ^5^			
Above HKD$7000	32(37.65)	61(71.76)	1
Below $7 K	48(56.47)	18(21.17)	5.08 (2.55–5.08)**
Presence of Unmanageable Debt	34(40.00)	6(7.06)	9.02 (3.53–9.02)**
Previous suicide attempt	43(50.59)	0(0.00)	-^c^
**Familial characteristics**			
Family history of suicide ^6^	20(23.53)	12(14.12)	1.87 (0.85–1.87)

^a ^The parameter estimates for each socio-demographic condition is controlled by age and gender.

^b ^Reference categories: ^1 ^Currently married or cohabited, ^2 ^above Form 3, ^3 ^living with others, ^4 ^currently employed, ^5 ^$7000 or more, and ^6^no family history of suicide

^c^ Unable to calculate owing to empty cell

*Note*. **Coefficient significant at *p *< .001

**Table 2:** Comparison of psychological, social environment, and life event characteristics of suicides and controls and unadjusted parameter estimates^a^

	Cases ^1 ^(N = 85)	Control ^1 ^(N = 85)	Odd Ratio (95% CI)
**Negative Life Events**			
Severity of Acute Negative Life Events ^b^			
Relationship	0.23 ± 0.79	0.01 ± 0.05	3.65 (0.72–18.52)
Family	0.02 ± 0.16	0.03 ± 0.16	0.92 (0.71–1.21)
Work or school	0.01 ± 0.06	0.05 ± 0.23	0.73 (0.43–1.24)
Physical health	0.08 ± 0.46	0.00 ± 0.00	50.13 (0.14–17684.07)
Legal issues	0.00 ± 0.02	0.00 ± 0.00	2.75 (0.64–20.34)
Severity of Chronic Negative Life Events ^c^			
Relationship	0.18 ± 0.54	0.09 ± 0.26	1.29 (0.90–1.86)
Family	0.13 ± 0.34	0.11 ± 0.38	1.03 (0.83–1.26)
Work or school	0.35 ± 0.78	0.16 ± 0.42	1.45 (0.98–2.13)
Physical health	0.08 ± 0.24	0.06 ± 0.28	1.13 (0.82–1.54)
Legal issues	0.10 ± 0.47	0.00 ± 0.00	1.17 (0.85–1.45)
**Sociological variables**			
Family Relationships			
Level of expressed emotions	19.97 ± 3.97	17.75 ± 2.42	2.06 (1.41–3.00)**
Extra-marital affairs^d^	4(7.69)	2(2.99)	3.02 (0.53–17.23)
Interpersonal Relationships			
Size of social support network	3.93 ± 2.96	7.35 ± 22.67	0.22 (0.03–1.43)
Frequency of social support	7.24 ± 5.19	8.50 ± 3.89	0.75 (0.54–1.06)
Social Support Content	3.34 ± 0.86	3.84 ± 0.33	0.30 (0.16–0.53)**
**Psychiatric Conditions**			
Psychiatric diagnosis	69(81.18)	10(11.76)	32.34 (13.75–76.06)**
Mood disorders	43(50.59)	10(11.76)	7.67 (3.50–16.83)**
Depression Score	23.10 ± 17.00	0.52 ± 2.80	91.44 (12.20–686.70)**
Ever received some kind of emotional treatment	38(44.71)	4(4.71)	16.52 (5.54–49.25)**
Ever received psychiatric treatment	27(31.76)	2(2.35)	30.43 (6.98–132.72)**
Presence of chronic physical illness	22(25.88)	9(10.59)	2.99 (1.29–6.98)
**Psychological Conditions**			
Impulsivity	4.58 ± 4.37	1.25 ± 1.52	5.31 (2.68–10.51)**
Social problem solving skills	20.60 ± 8.41	27.10 ± 5.89	0.38 (0.26–0.56)**
Healthy living styles	12.56 ± 3.74	15.01 ± 2.85	0.45 (0.31–0.65)**

^a ^The parameter estimates for each negative life event is controlled by age and gender.

^b ^Acute life events happened within 30 days prior death.

^c ^Chronic life events happened from a month to a year prior death.

^d ^Because of non-applicability reason, the total numbers of cases and controls do not add up to 85.

^1 ^These two columns report the n(%) for categorical variables and mean ± SD for continuous variables.

*Note*. **Coefficient significant at *p *< .001

### A multivariate logistic regression

All of the significant variables found from the binary logistic regression analyse were then further analysed by a multivariate logistic regression to find the most robust factors relating to suicide. Variables with significant values smaller than *p *< .001 were marital status, living arrangement, employment status, monthly income, presence of unmanageable debts, level of expressed emotions, social support content, psychiatric illness, history of emotional treatment, history of psychiatric treatment, impulsivity, social problem solving skill, and healthy living style. The multivariate analyses identified five independent significant risk factors for the middle-age suicides in Hong Kong. Table 3 presents the multivariate estimates on the five factors, namely, psychiatric illness, presence of unmanageable debts, currently unemployed or underemployed, never married, and living alone (all *p *< 0.05). The final model explains 71% of the proportion of the variance in between the control and suicide groups (Nagelkerke R Square = 0.71). Suicide risk increases strikingly with exposure to multiple risk factors: 100% (18/18) of those with four or more risk factors, 91.2% (31/34) of those with three risk factors, 63.6% (21/33) of those with two risk factors, and 33.3% (15/45) of those with one risk factor died by suicide. On the other hand, nearly half (40/85 = 47.1%) of the control subjects were free from the risks, 35.3% (30/85) of them presented one risk, 14.1% (12/85) presented two risk factors, and 3.5% (3/85) presented three risk factors. The sensitivity of the final model on completed suicide in the middle aged was therefore 100% (85/(85+0)) and the specificity 47.1% (40/(40+45)).

**Table 3:** Adjusted parameter estimates of risk and protective factors of suicide

	Adjusted Odd Ratio (95% CI)
Psychiatric Diagnosis	
No diagnosis	1.00
At least 1 diagnosis	37.55 (11.57–121.85)***
Presence of Unmanageable Debts	
No debt	1.00
Indebted	9.42 (2.18–40.83)**
Employment Status	
Currently employed	1.00
Unemployed or underemployed	4.81 (1.32–17.48)*
Marital Status	
Currently married or cohabited	1.00
Never married	4.19 (1.08–16.29)*
Living Arrangement	
Living with someone	1.00
Living alone	3.93 (1.16–13.36)*

***p < 0.001; **p < 0.01.; *p < 0.05

## Discussion

A surge in middle-aged suicides following the Asian economic recession has been observed in Hong Kong. This case-controlled psychological autopsy study examined a wide range of independent variables selected based on previous literature in both East and West. The data of the present study is based on the psychological autopsy study on 15–59-year-old [9]. However, because of the wide age-ranged of the subjects in the previous study, a masking effect of the non-linear relationship of age with other factors may occur. Thus, we report here the findings on suicides among the 30–49-year-old in order to improve our understanding of risk and protective factors of suicides among the middle-aged, and consequently would assist the development of prevention strategies.

Our multivariate analysis findings once again confirm the fact that suicides occur due to a complex interaction of socioeconomic factors (i.e., indebtedness and unemployment), social factors (i.e., never married and living alone), and psychiatric factors, especially the presence of mood disorders, on an individual maladaptive platform. Nevertheless, taken into account of the multiple factors being examined in the study, the strong relationship between socioeconomic problems and middle-aged suicides is particularly relevant to Asian context. The effect of adverse social-economic conditions on the middle-aged could be found to be serious or even fatal.

Previous studies have found relationships between unemployment, indebtedness, and suicides. However, the odd ratios for indebtedness and unemployment for our subjects were higher and at the higher end of other studies have reported. Previous controlled psychological studies conducted in Asian countries found that the ORs for unemployment for suicides ranged from 3.5 to 6.15 [17,23,24], and the ORs for unmanageable debts for suicide in Australia and in suicides who died by charcoal-burning in Hong Kong ranged from 2.8 to 6.38, respectively [25,26]. The considerable impacts of unemployment and indebtedness on suicides in the middle-aged Hong Kong Chinese could be understood from the traditional Chinese perspective and values on work and wealth among the Hong Kong Chinese. Work has always been closely connected with one's sense of self-worth in Chinese societies [27]. Chinese tend to define "self" primarily based on their role in salient social identities (i.e., husband, family provider, father etc.); thus, loss of job and fail to financially support oneself might be seen as a failure to fulfil such responsibilities. Consequently, discrepancy between actual performance and self expectations might trigger off depressive symptoms [28]. These cultural definitions on work and wealth were mainly possessed by males. Female suicides, however, may also be explained by the same values because cosmopolitan Hong Kong Chinese females seem to carry equal value on the importance of work and wealth when more of them participated in the labour-force.

Consistent with many (but not all) studies, lived alone [29,30] and never married [31-33] were found to be significant risk factors for the middle-aged suicides in our study. The convergence of socio-demographic effects on suicide, however, appears to vary across gender, cultures, and life span. For instances, an epidemiological study in Japan found that suicide rates were higher in prefectures where marriage was more common and divorce was less common [34]; a Pakistan study also revealed that more women committed suicide than did unmarried women [35]. These inconsistent findings suggest that the relationship between social factors and suicide is equivocal when cultural issues were taken into account. More research are needed to investigate the relationships between socio-demographic factors and suicide in different cultures and on an individual-level.

As in the majority of studies on completed suicide, the foremost predictor of dying by suicide is the presence of psychiatric illnesses [36,37]. This study shows that 81% of the Hong Kong middle-age suicides had been diagnosed with at least one psychiatric disorder. Mood disorders were found to be the most prevalent disorders among the cases (50.6%), which was in the mid range (29%–88%) reported in other psychological autopsy studies [36]. It is noteworthy that, apart from the presence of psychiatric illness, the severity of depressive symptoms was found to be a significant risk factor for suicide in the binary logistic regression analysis. This finding has a significant implication for suicide prevention. We agreed with Phillips and colleagues' [17] suggestion that suicide prevention in people with different types of mental illnesses could focus on the management of their depressive symptoms. However, to make this a sound and practical suicide prevention strategy in Hong Kong, we should take into consideration the relatively low awareness of depression in our community, the stigma of mental illness, and the poor access to psychiatric service that might hinder people's help-seeking behaviour. Thus, increasing public awareness about the treatment of psychiatric illnesses relevant to suicide, especially depression, and enhancing training of health professionals to detect depressive symptoms should be concomitantly implemented [38].

A few protective factors including social support, social problem-solving ability, and healthy living styles did not achieve statistical significance in the final multivariate regression model. However, they were found statistically significant as risk factors for middle-aged suicide. This finding was unexpected because in our 15–59-year-old analysis, social support was found to have a significant independent protective effect on suicide [9]. This unexpected finding could be related to the masking effect of a non-linear relationship between the factor of age and suicidal risk; and provide the support for conducting a separate analysis based on the middle-age group. Further research in exploring potential protective factors and examining their impacts on middle-aged suicides are needed.

### Limitations and future research

This study has its advantages, including its large and representative sample and its narrow studied age range. Yet, similar to many psychological autopsy studies, there are several limitations including its retrospective and cross-sectional nature, and heavy reliance on proxies' information [13,39-41]. There are two major limitations that should be acknowledged. First, though we have used both active (i.e., recruitment from public mortuaries by approaching the potential informants directly) and passive (i.e., recruitment from coroner's court via study introductory letter) recruitment methods, a low response rate may be considered as insufficient. However, given the fact that suicide is still regarded as a taboo in Hong Kong, a low response rate was anticipated. A relatively low response rate was also noted in some psychological autopsy studies in the UK [42,43] and the elderly suicide psychological autopsy study in Hong Kong [44]. Second, because of the case-control study design and the use of a logistic regression analysis, factors that only seen in subjects but not in controls could not be tested in the logistic regression model. For example, previous suicide attempts was not observed in the matched controls, thus it was excluded from the logistic regression analysis. This problem has been termed "complete separation" by Hosmer and Lemeshow [45]. Hence, although it has been well documented that a history of suicide attempts increases suicide risk [1,10,36,46-48], it failed to be identified as a robust risk factor in our study. Innovative statistical approaches are needed to deal with some of the special problems associated with the modelling of low base rate events such as completed suicide.

## Conclusion

This study suggests that at least one evidence-based intervention [8], which is raising the awareness in depression recognition and treatment in the community, seems to be an appropriate strategy to reduce middle-aged suicides in Hong Kong. Our results lean strong support to a multi-factorial approach to the understanding and prevention of suicide for the middle-aged by way of a range of multi-pronged and multi-levelled initiatives that aim at individual, family, and societal levels. Accordingly, we suggest that suicide prevention for the middle-aged might include raising public awareness of depression, enhancing the mechanism to detect early signs of depression, and improving treatment and management of depression; designing programmes to reduce social inequity and social discrimination against those who are unemployed, indebted, or socially isolated; and fostering good mental health skills to promote resiliency and addressing the psychosocial needs of those exposed to stress and adversity. The present findings might also be helpful to suicide prevention endeavourers in other countries, especially Asian countries, who are also subjected to the increasing trends of middle-aged suicides during the economic recession.

## Competing interests

The study was supported by the Hong Kong Jockey Club Charities Trust (which underwrote this research study via the Chief Executive's Community Project List 2002) and the Research Grant Council of Hong Kong (to Dr. Sandra Chan, CUHK 4373/03M; Project Code 2140401).

## Authors' contributions

PWCW and WSCC drafted the manuscript and were involved in the data collection and data analysis. EYHC, SSMC, YWL, and PSFY were involved in the design of the study. All authors were involved in the coordination of the data collection. PSFY had a leading role in the data analysis. All authors were involved in the interpretation and writing of the final manuscript.

## Pre-publication history

The pre-publication history for this paper can be accessed here:


